# Flashbacks in social anxiety disorder: Psychopathology of a case

**DOI:** 10.4103/0019-5545.43637

**Published:** 2008

**Authors:** Arthur Kummer, Estefania Harsanyi

**Affiliations:** Department of Internal Medicine – School of Medicine, Federal University of Minas Gerais, Brazil

**Keywords:** Social phobia, social anxiety disorder, flashbacks, psychopathology

## Abstract

Social anxiety disorder is characterized by overwhelming anxiety in everyday situations which are frequently avoided due to a fear of being watched and scrutinized by others or acting in an embarrassing way. Flashbacks are typical symptoms of post-traumatic stress disorder, and their main features are intrusive and vivid images that occur in a waking state. We present a case study of a man diagnosed with social anxiety disorder who had reexperiencing symptoms similar to flashbacks of what he considered “shameful situations”. The differential aspects between flashbacks and obsessional imagery are discussed. Reexperiencing symptoms and imagery of social phobia as well as the sociocultural influence over the symptomatology of psychiatric disorders are then highlighted.

## INTRODUCTION

Social anxiety disorder, also known as social phobia, is now well recognized as one of the most common psychiatric disorders. With an onset early in life, it is frequently impairing and associated with other anxiety disorders, depression, and substance use disorders.[1] Persons who have the generalized type of the disorder, fear or avoid most social situations, while people with the nongeneralized type of the disorder report anxiety or avoidance to limited performance situations.

Flashbacks are defined as involuntary, recurrent, intrusive and vivid images of a trauma that emerge in someone's mind in a waking state.[2] It is also conceptualized as re-experiencing part of a traumatic event with a realistic intensity as if it were happening in the present. These phenomena are one of the core symptoms of post-traumatic stress disorder (PTSD) and are sensitive and specific indicators of the presence of trauma.[2]

Although flashbacks are not pathognomonic of PTSD, these symptoms are only associated to PTSD and to drug-related phenomena. We present a singular case in which a young man with social anxiety disorder presents flashback(s) of what he considered “shameful situations”.

## CASE HISTORY

Mr. “A” is a single 26-year-old student, who attended our psychiatric unit with a complaint of severe anxiety in some social situations. Starting in his early teens, he noticed extreme anxiety when engaged in public speaking or socially approaching girls. He also reported a milder discomfort when talking to strangers and people in authority as well as speaking up in meetings. Despite feeling extremely anxious and fearing that he could act in a way which would embarrass himself, he insisted in enduring most of these situations. However, soon after this, he begins to censure himself for what he considered “shameful situations”. Curiously, he described repeated and distressing recollections, with realistic intensity, of what he considered his “worst moments”. He tried unsuccessfully to suppress these vivid images as they emerged involuntarily and intrusively. At these moments, he felt moderate psychological distress and mild physiological reactivity. These specific symptoms progressively waned within a few weeks and were replaced by other scenes of situations that he endured and described as “shameful moments”. They were similar to the flashbacks seen in PTSD, apart from the fact that Mr. A's description had an observer's perspective (i.e., seeing himself as if through the eyes of another person).

He received a diagnosis of social anxiety disorder according to DSM-IV criteria and engaged sessions of cognitive psychotherapy focusing on social anxiety disorder. Considering these flashbacks were fed by negative automatic thoughts related to social phobia, we expected that they would concomitantly ameliorate. The treatment aimed mainly at correcting dysfunctional negative self-appraisals and beliefs, and shifting the direction of his attention to the external aspects of the social environment, instead of his internal processes when undergoing the feared situations.

A progressive improvement in shyness and a decrease in anticipatory anxiety were noticed. His progress was less remarkable concerning dating but Mr. A was already satisfied with his development and interrupted his treatment. Of note, Mr. A confirms that the recurrent and intrusive images of his “shameful moments” have diminished significantly.

## DISCUSSION

We presented a case of a young man who presented intrusive sensorial phenomena with such realistic intensity which would meet current definitions of flashbacks.[2] There are some similarities between social phobia imagery and flashbacks in PTSD: both can be triggered by thoughts, physical sensations and external reminders; both are experienced as if they are occurring at the current time (nowness); and both are intrude and involuntary. One major difference between them is that social phobia flashbacks usually occur in the observer's perspective.[3]

In some cases, flashbacks may be confounded with obsessional imageries of obsessive-compulsive disorder. Lipinski and Pope described cases of obsessional imageries which were previously diagnosed as flashbacks of PTSD.[4] However, they are essentially different as the content of the obsessional images did not correspond to real past experiences of the patients and patients also had other obsessions and compulsions (e.g., cleaning or checking). Imageries of Mr. A were closely related to the exposure of feared situations and he had neither obsessions nor compulsions. Mr. A also could not meet DSM-IV PTSD criteria as the social events have not the traumatic intensity required by criterion A.[5] Considering that since Mr. A outbraved these fear-inducing situations, this could induce “small traumas” which were relived latter in a lighter way than what is seen in PTSD. Indeed, Erwin *et al.* have recently demonstrated that patients with social phobia with comorbid PTSD did not differ from patients without such comorbidity on reexperiencing, avoidance, and hyperarousal reported in connection with socially stressful events.[6]

Flashbacks are still a poorly understood phenomenon. Some electrophysiological and biochemical mechanisms have been enrolled,[7,8] but the amygdala is a pivotal structure in the pathophysiology of flashbacks.[9] The amygdala recognizes danger signals quicker than other brain structures and it is able to increase emotional valence before memory formation in order to make such information easier to access in future settings.[9] Interestingly, it has been suggested that flashbacks are not necessarily realistic copies of the traumatic events and are resulted from an exaggerated perception of trauma.[10] Thus, flashback formation may involve a hypersensibility of the amygdala to fear-inducing information. Furthermore, hyperexcitability of these fear circuits has also been related to the characteristic distorted cognitions of social phobia.[11]

However, flashbacks are strongly mediated by culture. It has been noticed that the frequency of flashbacks has increased substantially over time in soldiers subjected to the stress of combat.[12] Some authors have argued that a direct association between flashbacks and films exists, indicating that culture influence the expression of distressing memories.[12]

As the phenomenology of psychiatric disorders is culturally and historically determined, the case presented here indicates that the constellation of symptoms of social anxiety disorders may also be influenced by sociocultural factors.
